# An Investigation of the Effect of Attachment on Distress among Partners of Patients with Ovarian Cancer and Their Relationship with the Cancer Care Providers

**DOI:** 10.3390/curroncol28040258

**Published:** 2021-08-04

**Authors:** Danielle Petricone-Westwood, Jacqueline Galica, Sarah Hales, Elisa Stragapede, Sophie Lebel

**Affiliations:** 1 Department of Psychosocial Oncology, Tom Baker Cancer Centre, Alberta Health Services, 2202 2nd Street SW, Calgary, AB T2S 3C1, Canada; 2 Faculty of Health Sciences, School of Nursing, Queen’s University, Cataraqui Building, 92 Barrie Street, Kingston, ON K7L 3J8, Canada; jacqueline.galica@queensu.ca; 3 Department of Supportive Care, Princess Margaret Cancer Centre, University Health Network, 610 University Ave, Room 16747, Toronto, ON M5G 2C1, Canada; sarah.hales@uhn.ca; 4 School of Psychology, University of Ottawa, 136 Jean Jacques Lussier, Vanier Hall 4016, Ottawa, ON K1N 9A8, Canada; estra036@uottawa.ca (E.S.); sophie.lebel@uottawa.ca (S.L.)

**Keywords:** attachment, caregivers in cancer, ovarian cancer, distress

## Abstract

Caregivers of patients with ovarian cancer experience distress related to caregiving difficulties within cancer care. Attachment insecurity is a well-known protector of distress, particularly as it relates to support from others. Using multivariate analyses, this study sought to determine the contribution of attachment insecurity and experiences with cancer care on symptoms of depression and anxiety, and investigated whether attachment insecurity moderated the relationship between caregiving experiences and distress. Multiple hierarchical regression analyses were conducted as part of a larger cross-sectional questionnaire study of distress among partners of patients with ovarian cancer. Participants (*n* = 82) were predominantly male, white, had household incomes over $100,000 and postsecondary education. Caregiving experiences explained 56% of the variance in depression, and 28% of the variance in anxiety. Specifically, lack of time for social relations as a result of caregiving significantly predicted depression and anxiety. Attachment anxiety correlated with both depression and anxiety, but attachment avoidance did not. Neither attachment anxiety nor attachment avoidance significantly contributed to distress variance, and neither moderated any of the relationships between caregiving experiences and distress outcomes. This study highlights the importance for cancer care to recognize the effect of caregiving responsibilities upon caregivers’ mental health, regardless of vulnerability to distress.

## 1. Introduction

In their lifetime, one in two Canadians will be diagnosed with cancer [1], meaning a significant portion of Canadians will act as informal caregivers to these patients. As cancer care has advanced to outpatient treatment settings, the healthcare system has grown to depend on cancer caregivers to support the patient and ensure treatment adherence [2]. These caregivers often prioritize their ill loved one’s health needs over their own, including their mental health.

It is well documented in the literature that cancer caregivers experience significant distress [3], including symptoms of depression and anxiety, and other compromises to their quality of life [3,4]. A recent meta-analysis reported prevalence rates for depression at 42% and anxiety at 47% among caregivers. They reported that spouse-caregivers, avoidant caregivers, and younger caregivers were at greater risk of depression [3]. Investigations on the caregiving experience have found the relationship with healthcare providers (HCP) as important and influential for caregivers [5]. Caregivers often report a lack of information and resources [5,6], often related to their loved one’s cancer, the provision of comfort and support, and how to maintain their own wellbeing [7].

Caregivers of patients with ovarian cancer are understudied, and face significant challenges as ovarian cancer is mostly diagnosed at a late stage, frequently recurs, and has a poor prognosis [8,9]. Partner-caregivers are often men, who are underrepresented in the caregiver literature [10]. Their distress is often underreported [11] and underestimated by HCP [12].

Attachment is a highly studied construct that is well established in psychological literature and practice to influence emotion regulation, distress related to stressful events, and mental health [13]. Attachment theory posits that when someone is faced with a threat, their reactions are shaped by early relationships with parental or caregiving figures [14,15]. Attachment is conceptualized as the intersection of two main working models: (1) the view of self (worthiness of love and care), and (2) the view of others (reliability and availability of others to provide care). For measurement purposes, the working models can be conceptualized into two main dimensions of attachment insecurity: (1) attachment anxiety, related to a negative view of self, including a fear of rejection and beliefs of being unworthy of care, and (2) attachment avoidance, related to a negative view of others, including an avoidance of dependence on others and of intimacy [16]. Attachment can be measured through self-report as either romantic attachment, pertaining to romantic relationships, or general attachment oriented towards all relationships [17].

Investigations have found that insecure attachment can result in a perceived lack of support from attachment figures and a perception that they are less responsive [18]. Higher levels of general and romantic attachment anxiety and attachment avoidance have both been found to predict higher levels of depressive symptoms, anxiety, and a lower perceived social support among cancer caregivers [19]. Attachment needs are activated when there is the presence of a threat [20], which has been widely studied in cancer [19,21,22]. Additionally, HCP relationships with patients are influenced by the patient’s attachment [23]. It has yet to be investigated, however, whether attachment insecurity amongst caregivers contributes to the relationship between their caregiving experiences and distress. As attachment has an influence on perceived support and responsiveness, attachment insecurity among caregivers may increase distress through appraisal of the HCP as less supportive or dependable.

### Objectives

The authors recently conducted a cross-sectional exploratory study of partners of patients with ovarian cancer, which investigated a broad range of caregiving experiences within the cancer care system. Using descriptive and univariate analyses, the study evaluated the prevalence of those caregiving experiences and explored whether they correlated with symptoms of depression and anxiety [24]. Five caregiving experiences correlated with either depression or anxiety: (1) having a higher caregiving workload, (2) needing more help from HCP, (3) having problems with the quality of communication and information from HCP, (4) lacking information from HCP, and (5) lacking time for social relations as a result of caregiving.

The current investigation seeks to understand what mechanisms might link these caregiving experiences with more distress. Using an attachment framework and multivariate analyses, the current study sought to determine the role of general attachment insecurity on caregiving experiences of cancer care and ovarian cancer caregivers’ distress using the same sample from the original publication. It additionally sought to determine whether this moderated the correlations between distress and experiences within the healthcare system, identified in the previous analysis. The hypotheses were that among partner-caregivers of individuals with ovarian cancer:

1. Higher levels of attachment anxiety would be correlated with higher scores of symptoms of depression and anxiety;
2. Higher levels of attachment avoidance would be correlated with higher scores of symptoms of depression and anxiety;
3. Attachment anxiety levels would moderate the known relationships between the variables of experiences with cancer care and HCP, and symptoms of depression and anxiety. Specifically, higher levels of attachment anxiety would relate to a stronger relationship between experiences with cancer care and symptoms of depression and anxiety;
4. Attachment avoidance levels would moderate the known relationships between the five variables of experiences with cancer care and HCP, and symptoms of depression and anxiety. Specifically, higher levels of attachment avoidance would relate to a stronger relationship between experiences with cancer care and symptoms of depression and anxiety.

## 2. Materials and Methods

### 2.1. Study Design

This study’s protocol was approved by the Research Ethics Boards (REB) of the University of Ottawa (#H05-17-02), Queen’s University (#NURS-455-18), and the University Health Network (#18-5213). This exploratory analysis was part of a larger a cross-sectional, correlational study investigating partner-caregivers of patients with ovarian cancer and their relationships with HCP. See Petricone-Westwood et al. (2021) for the original publication for study design and detailed recruitment method [24].

### 2.2. Recruitment

Partner-caregivers of individuals diagnosed with any stage of ovarian cancer were invited to participate if: (1) the patient had been diagnosed with, treated for, or had recurrence of ovarian cancer within the last 5 years, (2) partner-caregivers were over 18 years of age, (3) they spoke and read in English or French, (4) they had met the cancer care team. 

Recruitment efforts involved the circulation of advertisements through Ovarian Cancer Canada, including through their national and regional newsletters, and through their social media (October 2017 to August 2019). Advertisements were posted in cancer survivorship centres across Canada, and through support groups in Kingston, Ontario and Calgary, Alberta. Active recruitment was conducted through gynecology oncology centres at the Princess Margaret Cancer Centre of the University Health Network in Toronto, Ontario (April 2019 to August 2019) and through Cancer Centre of Southeastern Ontario in Kingston, Ontario (January 2019 to August 2019). Participants were approached through identified patients with ovarian cancer who consented to us contacting their partner-caregivers. Verbal consent was obtained when the primary author screened participants. Consent forms were provided with the questionnaires, and informed consent was provided in returning completed questionnaires.

### 2.3. Measures

#### 2.3.1. Sociodemographic Variables

Sociodemographic variables were collected from participants. They were asked to report their understanding of medical variables related to their partners’ cancer. This included the date of diagnosis of the ovarian cancer, stage of the ovarian cancer, and which treatments patients had received for the cancer (i.e., surgery, chemotherapy, radiation, or combinations of these three).

#### 2.3.2. Caregiving Experiences with HCP

The Cancer Caregiving Tasks, Consequences, and Needs Questionnaire (CaTCoN) was used to evaluate multiple facets of the caregiving experiences as part of the healthcare system. The scale measures consequences of caregiving and relationships with the patient’s HCP [25]. For the purposes of this investigation, five of the nine subscales that correlated with depression or anxiety symptoms in the previous analyses were used [24]. They were (1) lack of time for social relations as a result of caregiving, (2) lack of information from HCP, (3) problems with the quality of information and communication from HCP, (4) needing more help from HCP, and (5) caregiver workload. These subscales are interpreted separately (i.e., there is no total score). Subscale items are Likert-style items as well as yes/no items. Likert-style items are scored from 0 (not a concern nor unmet need) to 3 (high concern or unmet need). Yes/no items are scored either 0 (not a concern) or 1 (a concern). These items were transformed to be out of 100, and a mean score was attributed for each subscale. The authors recommend only scoring weight-baring items, and subscale scores are continuous [25].

#### 2.3.3. Depression and Anxiety

The Hospital Anxiety and Depression Scale (HADS) [26] was used to measure symptoms of depression and anxiety. This 14-item scale yields two separate subscales, depression (HADS-D, seven items) and anxiety (HADS-A, seven items), and has been validated for use among cancer caregivers [27,28]. The scale items are continuous and yield total subscale scores from 0 (no distress) to 21 (high distress). Scores 8 to 10 were considered indicative of subclinical distress, and 11 and above indicative of clinical distress [26].

#### 2.3.4. Attachment Insecurity

A modified version of the Experiences in Close Relationships Scale (ECR-12) [29] was used to measure general attachment insecurity. The scale has two continuous subscales, attachment avoidance and attachment anxiety. The ECR-12 scale has demonstrated strong psychometric properties in its romantic attachment version [29]. Subscale scores are calculated through the mean Likert-item response, ranging from 1 (low/no attachment insecurity) to 7 (high attachment insecurity). The scale items were modified according to published instructions to measure general attachment [17,22]. For example, the item “I feel comfortable depending on romantic partners” which was modified to “I feel comfortable depending on others.” One item was not converted to the general prompt and was discarded (item: “I tell my close relationship partners just about everything”). Another item was discarded following an exploratory factor analysis as it did not adequately load onto the attachment anxiety subscale (item: “I worry a fair amount about losing others”), leaving a total of 10 items [30].

### 2.4. Analyses

Data was entered and analyzed using SPSS version 26. A simple imputation was conducted as only 1.14% of data were missing. Descriptive statistics were conducted on all sociodemographic, medical, predictor and outcome variables. Correlational analyses were conducted on all sociodemographic and medical variables to identify confounds. Two-tailed independent-sample t-tests were used to compare any significantly differing sociodemographic categorical variables. Pearson correlational analyses were conducted to test for correlations between attachment variables, caregiving experiences subscales, and distress subscales. 

Multiple hierarchical regression analyses were used to determine the explained variance of both attachment insecurity variables and variables related to caregiving experiences in cancer care on symptoms of depression and anxiety. Identified sociodemographic and medical covariates were entered in the first block, and attachment avoidance and anxiety were entered in the second block. In the final block, the five variables related to caregiving experiences were entered: caregiver workload, needing more help from HCP, lack of information from HCP, problems with the quality of information and communication with HCP, and lack of time for social relations.

Multiple hierarchical regression analyses were also used to test for moderation and the explained variance of covariates, independent variables, and interaction effects of moderators. Attachment anxiety and attachment avoidance were separately tested as moderators between both outcome variables (symptoms of depression or anxiety), and each CaTCoN subscale as the predictor variable. Any sociodemographic or medical variables that correlated with outcome variables were controlled for as covariates in the first step of the hierarchical regression. 

Hayes and Rockwood [31] argue that moderating variables do not have to be statistically related to independent variables, as per Baron and Kenny’s [32] initially proposed assumptions. Moderation models of attachment were deemed appropriate to test regardless of statistical significance of Pearson correlations between moderating variables and the predictor and outcome variables [31]. This resulted in multiple separate moderation analyses. This was judged to be optimal for hypothesis testing, as the subscales were not able to be combined into a single variable of distress nor of the caregiving experience. The two HADS subscales do not yield adequate psychometric properties for a total HADS score [27], and CaTCoN subscales were only correlated at a range of *r* = 0.24 to 0.62, suggesting they could not be combined into one variable.

A significance level of 0.05 and confidence intervals of 95% were employed to determine significance. As 20 separate analyses were conducted, a Bonferroni correction was applied to the *p*-value to reduce the risk of Type 1 error when interpreting our hypotheses (multivariate analyses). As such, a *p*-value of 0.0025 was used to determine significance of our novel analyses. Pearson correlations were interpreted using Cohen’s [33] method, where an *r*-value smaller than 0.29 was considered small, 0.30 to 0.49 were considered moderate, and 0.5 and over were considered strong [33].

## 3. Results

For adequate reporting and clarity, previously published data on sociodemographic information, prevalence of depression and anxiety symptoms, and correlations with caregiving experiences are reported again in this paragraph, in the correlative analysis section, and in Table 1 [24].

Participants (*n* = 82) were 97.5% men, with a mean age of 57.2 (*SD* = 12.1) years. These participants were mostly white (89.9%), and were English-speaking (91.5%). Half of participants had a total household income over $100,000 (52.5%), with others reporting $20,000 to $40,000 (7.5%), $40,000 to $60,000 (11.3%), $60,000 to $80,000 (16.3%), and $80,000 to $100,000 (12.5%). Participants’ highest level of education varied from high school diploma (12.2%), some postsecondary (22%), postsecondary degree (29.3%), some postgraduate (7.3%), or postgraduate degrees (25.6%). The average length of relationship with their partners with ovarian cancer was of 28.5 (*SD* = 14.8) years. Their partners living with ovarian cancer were diagnosed on average 20.8 (*SD* = 28.6) months prior to the study completion. Most partners were diagnosed at Stage III (53.9%) or IV (21.1%), and fewer at stage I (13.2%) or II (11.8%). A large majority of patient-partners were treated with both surgery and chemotherapy (80.2%). Most participants were recruited through active recruitment at the Princess Margaret Cancer Centre (56.1%), and Cancer Centre of Southeastern Ontario (17.1%), and a smaller portion responded to national advertisements (26.8%).

### 3.1. Correlative Analysis

#### 3.1.1. Depression and Anxiety

The mean symptoms of depression score was of 5.0 (*SD* = 4.3). In the sample, 8.5% had clinical levels of depression and 23.2% were subclinical. Symptoms of depression was not significantly correlated with any sociodemographic or medical variables [24], see Table 1. Depression was strongly correlated with caregiving workload (*r* = 0.6, *p* < 0.001), lack of time for social relations (*r* = 0.7, *p* < 0.001), lack of information from HCP (*r* = 0.5, *p* < 0.001); moderately correlated with needing help from HCP (*r* = 0.5, *p* < 0.001). Depression was weakly correlated with problems with the quality of information from HCP (*r* = 0.3, *p* = 0.02 [24], however this would no longer be significant with a Bonferroni correction as *p* > 0.0025. See Table 1 for all outcomes.

Participants’ average anxiety score was 7.1 (*SD* = 4.2). Twenty three percent of the sample were in the clinical range of anxiety, and 20.7% were subclinical [24]. Anxiety was weakly negatively correlated with age (*r* = −0.3, *p* = 0.02), and higher anxiety was found among participants who responded to advertisements to participate, as opposed to those who were identified through active recruitment (*M_Adver_* = 8.9, *SD_Adver_* = 4.4, *n_Adver_* = 22 vs. *M_ActRec_* = 6.5, *SD_ActRec_* = 4.0, *n_ActRec_* = 60; *p* = 0.018) [24]. Using partial correlations with these variables, anxiety was strongly correlated with lacking time for social relations (*r* = 0.6, *p* < 0.001), and moderately correlated with caregiving workload (*r* = 0.4, *p* < 0.001), lack of information from HCP (*r* = 0.3, *p* = 0.01), and needing help from HCP (*r* = 0.3, *p* = 0.04). Again, with a Bonferroni correction, however, some of these correlations would no longer be considered significant. Anxiety was not correlated with having problems with the quality of communication and information from HCP, nor with other sociodemographic or medical variables [24] (see Table 1).

#### 3.1.2. Attachment

The mean score for attachment avoidance was of 4.3 (*SD* = 1.2, *min* = 1.4, *max* = 7.0) and for attachment anxiety was of 3.0 (*SD* = 1.1, *min* = 1.0, *max* = 5.6). Cut-offs for elevated levels of attachment insecurity have been established for romantic attachment versions of the ECR-36 [34]. According to the cut-off of 2.5, 90.2% of the sample had elevated attachment avoidance. The cut-off of 3.5 for attachment anxiety [34] indicates that 34.1% of the sample reported elevated attachment anxiety. These two subscales were not significantly correlated with one another (*r* = −0.1; *p* = 0.35). Without the Bonferroni correction, attachment anxiety was correlated with household income (*ρ* = −0.3, *p* = 0.006), depression (*r* = 0.3, *p* = 0.01), anxiety (*r* = 0.3, *p* = 0.008), and was significantly lower among participants whose partners had chemotherapy (*t*(79) = 3.1, *p* = 0.003; *M_Chemo_* = 2.9; *SD* = 1.1, *n* = 76 vs. *M_NoChemo_* = 4.4; *SD* = 0.7, *n* = 5). Attachment anxiety was not significantly correlated with the CaTCoN subscales. Attachment avoidance was not significantly correlated depression, anxiety, nor CaTCoN subscales.

### 3.2. Regression Analyses

#### 3.2.1. Depression

The linear regression model including attachment insecurity and all CaTCoN subscales on depression was significant overall and explained 65.1% of the variance in depression (*F*(7,73) = 19.4, *p* < 0.001, *R*^2^ = 0.7), see Table 2. Attachment insecurity variables predicted 8.7% of the variance of depression. Neither attachment anxiety nor attachment avoidance significantly contributed to the model when applying a Bonferroni correction. Variables related to the caregiving experience explained 56.4% variance. Of these five variables, only reporting a lack of time for social relations significantly contributed to the model. Reporting a lack of information from HCP, having a higher caregiver workload, needing more help from HCP and having problems with the quality of information and communication with HCP did not significantly contribute to depression.

#### 3.2.2. Anxiety

The linear regression model analyzing attachment insecurity and all CaTCoN subscales on anxiety was significant overall and explained 49.8% of the variance in anxiety (*F*(9,70) = 7.7, *p* < 0.001, *R*^2^ = 0.5), see Table 2. Covariates explained 10.7% of the variance in anxiety. Neither recruitment nor age significantly contributed to the model. Attachment insecurity variables contributed an additional 10.9% of the variance of anxiety, however this was not significant with Bonferroni correction and neither variables significantly contributed to the model. The caregiving experiences variables explained 28.2% of the variance. Among these five variables, only having a lack of time for social relations due to caregiving significantly contributed to the model. The other four variables did not significantly contribute to the model predicting anxiety.

### 3.3. Moderation Analyses

Attachment anxiety and attachment avoidance did not significantly moderate any of the relationships between depression or anxiety and needing help from HCP, lack of time for social relations, caregiver workload, problems with the quality of information and communication from HCP, and lacking information from HCP. 

## 4. Discussion

This investigation sought to better understand how caregiving experiences, general attachment anxiety, and general attachment avoidance contribute to symptoms of depression and anxiety among partner−caregivers of patients with ovarian cancer. This analysis built on published findings that established a connection between the caregiver’s role as part of the cancer care system and their distress [24]. The hierarchical regression models were significant. In models of both depression and anxiety, only lacking time for social relations due to caregiving responsibilities significantly contributed to a portion of the variance among partners of patients with ovarian cancer. Caregiving workload and lacking information, needing more help from the HCP and having problems with the quality of information and communication with HCP did not significantly predict either depression or anxiety. Comparatively, our univariate analyses found a significant relationship between caregiving workload, having a lack of information, needing more help from HCP, and having problems with the quality of communication with HCP. These findings suggest that care demands may prevent caregivers from employing coping strategies such as turning towards their friends and family.

General attachment insecurity did not significantly contribute to our model of distress, which is noteworthy when we consider the general literature on attachment. Both general and romantic attachment insecurity are widely considered indicators and predictors of poorer mental health and coping [13,35], including among cancer patients and caregivers [19]. This is particularly true when considering the relationship between distress and social support [19]. Considering this, these results suggest that individuals who might typically have lower distress due to low levels of insecure attachment, may still experience distress depending on the context of their caregiving. In other words, all caregivers are vulnerable to distress if caregiving demands prevent them from accessing their typical support systems of friends and family.

These results provide support to the *wear and tear hypothesis* of caregiving [36,37], which posits that caregivers experience poorer health and mental health outcomes due to the long-term, unabated demands of caregiving. This study suggests that while indicators of resilience such as low attachment insecurity typically act as a protective factor, the demands of caregiving likely are detrimental to the caregiver’s mental health, particularly as their partner’s ovarian cancer progresses. This study provides a nuanced interpretation of the needs of ovarian cancer partner-caregivers as they pertain to their attachment, and provide further justification for preventative, practical interventions in addition to psychosocial intervention for caregivers that often target attachment insecurity.

### Limitations and Future Directions

While our study employed a Bonferroni correction to account for multiple analyses, this may have resulted in Type II error, given our conservative *p*-value combined with a small sample size. Additionally, this study’s sample was biased being mostly white, affluent and educated men. Future research would benefit from further efforts to recruit marginalized caregivers, who likely experience more barriers in their caregiving roles. Further, the direction of the relationship is to be considered, as it is possible that it is distress that leads to poorer appraisals of their caregiving experiences. Depending on the gynecology oncology centre where their ill partners were treated, it is possible also that there were multiple oncologists or residents involved in care, creating further variability in who caregivers considered when responding to CaTCoN items related to their relationships with HCP.

There were no specifications around the wife’s current health status (i.e., having a recurrence, active treatment, palliation, remission), or specific timeframes on the CaTCoN, which may have varied caregiver responses. Future investigations may collect more detailed information on these phases of treatment and the patient’s current health status and functioning, as this may influence both demands on the caregivers and their distress. The data additionally were subjective responses from the caregiver’s perspective, and may have differed if data were collected through other means such as through the patients or HCP, or objective measures such as referrals, clinic attendance and use of hospital resources. Future investigations may evaluate external factors such as the environment of caregivers, rotating HCP versus specifically assigned HCP, or to look at objective data such as referrals and interactions in clinic.

As a cross-sectional investigation, this study only captured a “snapshot” in time for each caregiver, but their experiences likely evolved throughout the cancer trajectory [38]. Future investigations may evaluate how relationships with HCP evolve overtime, particularly in ovarian cancer where there are frequent recurrences and poorer prognoses. 

This study yielded some unusual findings in attachment, suggesting attachment outcomes should be interpreted with caution, particularly given the limited reliability and generalizability of our convenience sample. For one, attachment anxiety and attachment avoidance were not correlated with one another. These constructs are theoretically orthogonal, however typically there is at least a low level of correlation found [39]. Additionally, 90% of this sample reported clinical levels of attachment avoidance, which was not correlated with distress. 

## 5. Conclusions

This investigation has further elucidated the high levels of distress experienced by mostly male partners of patients with ovarian cancer as it pertains to both their attachment insecurity, and their experiences with HCP. As the healthcare system continues to depend on caregivers, it is important to consider how this dependence may lead to an increase in caregiver distress and compromises to their quality of life. While intrapersonal considerations, such as attachment insecurity, are often considered in determining vulnerability to distress, this analysis suggests that it essential to consider how the healthcare team and cancer care as a system can support these caregivers, regardless of caregiver’s psychological vulnerability to distress. Additionally, while psychotherapeutic interventions that target attachment are known to improve distress [40], such interventions may not be sufficient nor realistic given the demands placed on caregivers in supporting their loved ones with cancer.

## Figures and Tables

**Table 1:** Correlations between participant sociodemographic and patient medical variables with distress, attachment insecurity, and caregiving experiences and consequences outcomes.

Variables and Outcomes	HADS		ECR		CaTCoN				
	Depr.	Anx.	Avoid.	Anx.	Social Relat.	Need Help	Work-Load	Lack Info.	Comm. Problems
Sociodemographic variables									
Age	−0.15	−0.26 *	0.10	−0.02	−0.08	0.03	−0.02	−0.22 *	−0.028 *
Education	0.06	−0.02	−0.08	−0.08	0.13	0.04	−0.14	0.29 **	0.07
Income	0.10	−0.08	0.03	−0.30 *	0.05	−0.01	−0.02	0.27 *	0.06
Length of relationship	−0.19	−0.04	0.02	−0.14	−0.05	0.04	−0.19	−0.14	−0.26 *
Medical variables									
Time since diagnosis	−0.05	0.03	−0.00	0.00	0.04	0.11	−0.07	−0.01	−0.06
Stage of ovarian cancer	0.07	0.07	0.08	0.02	0.14	0.16	0.20	−0.15	−0.20
HADS									
Depression	—	0.78 **	0.11	0.31 **	0.72 **	0.53 **	0.56 **	0.52 **	0.26 *
Anxiety	0.78 **	—	0.09	0.37 **	0.59 **	0.45 **	0.37 **	0.38 **	0.26 *
ECR									
Avoidance	−0.09	−0.11	—	0.10	−0.12	−0.08	0.11	−0.09	−0.17
Anxiety	0.31 **	0.37**	−0.07	—	0.14	0.20	0.17	0.06	0.11
CaTCoN									
Social Relations	0.72 **	0.59 **	0.10	0.14	—	0.48 **	0.56 **	0.49 **	0.24 *
Need Help HCP	0.47 **	0.33 *	0.10	0.20	0.48 **	—	0.37 **	0.56 **	0.29 **
Workload	0.57 **	0.37 **	−0.10	0.17	0.56 **	0.37 **	—	0.28*	0.08
Lack information	0.52 **	0.31 *	0.02	0.06	0.49 **	0.56 **	0.28 *	—	0.62 **
Problems with communication	0.26 *	0.16	0.13	0.11	0.24 *	0.29 **	0.08	0.62 **	—

*Note.* Categorical variables analyzed with categories listed in text; no Bonferroni correction applied to this data * p < 0.05. ** *p* < 0.01.

**Table 2:** Hierarchical regression models for attachment insecurity, and caregiving experiences in cancer care as predictors of symptoms of depression and anxiety.

Steps and Predictors	Depression					Anxiety				
	*β*	*R* ^2^	∆*R*^2^	*Ptl* ^a^	*Prt* ^a^	*β*	*R* ^2^	∆*R*^2^	*Ptl* ^a^	*Prt* ^a^
Step		—	—				0.11	0.11		
Age	—			—	—	−0.21			−0.21	−0.20
Recruitment type	—			—	—	−0.22			−0.23	−0.22
Step		0.09	0.09				0.22	0.11		
Attachment anxiety	0.29			0.29	0.29	0.33			0.34	0.32
Attachment avoidance	0.09			0.09	0.09	0.15			0.16	0.14
Step		0.65 *	0.56 *				0.50 *	0.28 *		
Problems with the quality of information and communication with HCP	−0.09			−0.12	−0.07	−0.04			−0.04	−0.03
Lack of information from HCP	0.24			0.24	0.15	−0.02			−0.02	−0.01
Caregiving workload	0.20			0.26	0.16	0.03			0.03	0.02
Lack of time for social relations	0.45 *			0.48	0.32	0.43 *			0.40	0.31
Need more help from HCP	0.10			0.13	0.80	0.20			0.21	0.15

*Note.* Age and recruitment type were only covariates in the anxiety model, and this were only included in the analysis with anxiety as an outcome. ^a^ *Ptl* = Partial correlations, *Prt* = Part correlations. * *p* < 0.001.

## Data Availability

The data presented in this study are available on request from the corresponding author. The data are not publicly available due to privacy.
